# Utilization of synthesized silane-based silica Janus nanoparticles to improve foam stability applicable in oil production: static study

**DOI:** 10.1038/s41598-023-46030-1

**Published:** 2023-10-30

**Authors:** Amir Hossein Saeedi Dehaghani, Reza Gharibshahi, Mohammad Mohammadi

**Affiliations:** Faculty of Chemical Engineering, Tarbiat Modares University, Tehran, Iran

**Keywords:** Nanoparticles, Synthesis and processing, Chemical engineering

## Abstract

This study investigated the effect of silane-based silica (SiO_2_) Janus nanoparticles (JNPs) on stabilizing the foam generated by different types of gases. Two types of SiO_2_ JNPs were synthesized through surface modification using HMDS and APTS silane compounds. Static analyses were conducted to examine the impact of different concentrations of the synthesized nanoparticles in various atmospheres (air, CO_2_, and CH_4_) on surface tension, foamability, and foam stability. The results indicated that the synthesized SiO_2_ JNPs and bare SiO_2_ nanoparticles exhibited nearly the same ability to reduce surface tension at ambient temperature and pressure. Both of these nanoparticles reduced the surface tension from 71 to 58–59 mN m^−1^ at 15,000 ppm and 25 °C. While bare SiO_2_ nanoparticles exhibited no foamability, the synthesis of SiO_2_ JNPs significantly enhanced their ability to generate and stabilize gas foam. The foamability of HMDS-SiO_2_ JNPs started at a higher concentration than APTS-SiO_2_ JNPs (6000 ppm compared to 4000 ppm, respectively). The type of gas atmosphere played a crucial role in the efficiency of the synthesized JNPs. In a CH_4_ medium, the foamability of synthesized JNPs was superior to that in air and CO_2_. At a concentration of 1500 ppm in a CH_4_ medium, HMDS-SiO_2_ and APTS-SiO_2_ JNPs could stabilize the generated foam for 36 and 12 min, respectively. Due to the very low dissolution of CO_2_ gas in water at ambient pressure, the potential of synthesized JNPs decreased in this medium. Finally, it was found that HMDS-SiO_2_ JNPs exhibited better foamability and foam stability in all gas mediums compared to APTS-SiO_2_ JNPs for use in oil reservoirs. Also, the optimal performance of these JNPs was observed at a concentration of 15,000 ppm in a methane gas medium.

## Introduction

In today's context, the decrease in natural oil production from reservoirs has highlighted the necessity for implementing new methods to either increase or sustain production levels^1^. To date, various techniques collectively known as enhanced oil recovery (EOR) methods have been employed^2–4^. Many of these methods involve the injection of water, gas, and various chemicals into oil reservoirs. Among these, gas injection stands out as a crucial and practical EOR approach^5,6^. Gas injection is economically advantageous due to its widespread availability and, unlike water injection, it avoids issues such as scaling, rising aquifer levels, bacterial growth, and similar concerns. Typically, associated gas is reinjected into the reservoir to maintain the pressure necessary for continuous oil production. However, the key challenge in gas injection lies in controlling the mobility of the injected gas, given its high mobility, which poses significant challenges for its injection and movement within the reservoir^7,8^.

When gas is injected into oil reservoirs, several issues may arise within the reservoir itself, including the fingering phenomenon, gravity override, and channelization (especially in fractured carbonate reservoirs). Due to its high mobility, gas rapidly reaches the production zone during injection, ultimately resulting in only the injected gas being recovered from the production well after some time^9^. To address these challenges, foam formation has emerged as a highly practical and cost-effective method for controlling mobility and enhancing apparent viscosity^10^. Foam can be generated using water, gas, and a surfactant as stabilizers. Injecting foam into the porous medium can improve the mobility ratio of the injected fluid, enabling better sweep efficiency and increased oil recovery from a larger reservoir surface area^11,12^.

While foam is widely accepted for EOR, certain issues hinder its practicality. Foam is typically generated using surfactants, and as a result, it can become unstable in oil reservoirs when exposed to high pressure and temperature. Therefore, modification techniques, such as the use of nanoparticles and polymers, become necessary to stabilize foam under the actual conditions of oil reservoirs^10^. In such circumstances, nanoparticles play a crucial role in preventing foam collapse and altering the wettability of reservoir rock from oil-wet to strongly water-wet^13^.

However, the strong van der Waals forces interactions among nanoparticles present a technical challenge for their colloidal stability in the base fluid^14,15^. One of the most commonly employed nanoparticles in the EOR process is SiO_2_ nanoparticles, extensively used to enhance foam stability^16–20^. SiO_2_ nanoparticles are favored in EOR experiments due to their availability and unique physio-chemical properties^21^. They are cost-effective and highly compatible with oil reservoirs, minimizing formation damage and destructive interactions with reservoir rock. SiO_2_ nanoparticles exhibit remarkable mechanical and chemical resistance, even under harsh oil reservoir conditions, including high temperature, pressure, and salinity^22^. They enhance foam stability by reducing the rate of liquid drainage and increasing maximum capillary pressure. Being inherently hydrophilic and highly reactive, SiO_2_ nanoparticles can establish strong bonds with various substances, particularly water molecules^23^. By fine-tuning the hydrophilicity of the SiO_2_ nanoparticles' surface, these particles can reduce interfacial surface tension (IFT) and extend the stability of generated foam for several days by residing at the interface between the water and gas phases^24,25^. To enhance the hydrophobicity of SiO_2_ nanoparticles, silane materials can be employed as coating agents^26^. This coating layer results in particle accumulation in the water phase, leading to increased viscosity and, consequently, a significant improvement in foam stability^27^. Previous studies have demonstrated that SiO_2_-stabilized foams in different gases can recover more oil from porous media compared to surfactant-stabilized foam^28,29^.

With the recent advancements in nanoparticle surface modification techniques, the use of JNPs as a novel strategy to enhance foam efficiency in oil reservoirs has garnered attention^30^. JNPs have gained widespread use in recent years due to their distinctive properties for stabilizing gas–water emulsions^31–33^. Unlike common nanoparticles, JNPs exhibit two or more distinct characteristics. For example, the simplest JNPs feature both a hydrophobic and a hydrophilic part^34,35^. These JNPs possess significantly higher adsorption free energy compared to conventional nanoparticles, resulting in greater interfacial activity and improved adsorption stability. These attributes enhance the stability of gas foam, particularly under high-temperature and high-pressure conditions^30^. JNPs can be synthesized using methods such as masking, bottom-up assemblies, and controlled phase separations, with the masking method being the simplest and quickest^36^. These nanoparticles have the capability to stabilize gas–water emulsions for extended periods^37,38^.

Operational and reservoir conditions also play a pivotal role in the effectiveness of an EOR process^39^. Temperature is a primary factor influencing foam efficiency. Research has shown that at elevated temperatures, the surface tension between gas and water increases, leading to enhanced particle mobility and separation of surface-active ingredients^40^. Pressure is another significant factor; an increase in pressure reduces the surface tension between gas and water, consequently improving foamability. However, higher pressure also results in increased gas solubility in the water phase, decreased water viscosity, and reduced foam stability^41^. It is also anticipated that foam stability will improve with an increase in the concentration of JNPs up to an optimal level. Additionally, the type of gas used appears to have an impact under real conditions. Previous findings have indicated that the performance of N_2_ gas foam surpasses that of CO_2_, highlighting the importance of gas selection^40,42^.

Despite the numerous studies conducted, the utilization of these nanoparticles presents various challenges that researchers must address. There is a need for further investigation into surface modification methods and the optimal synthesis of JNPs. Additionally, the potential of methane (CH_4_) gas, readily available but not yet explored, as a suitable medium for foam production with the assistance of JNPs should be examined. In general, the impact of gas type on foam properties, encompassing foamability and foam stability of JNPs, remains unexplored.

Therefore, this study comprises two main components. The first part aims to synthesize SiO_2_ JNPs using two different silane agents, namely HMDS and APTS. Characterization of the synthesized JNPs involves various analyses, including FTIR, DLS, and SEM. To assess the colloidal stability of nanoparticles and their resistance to aggregation, Zeta potential measurements were conducted. In the context of gas injection processes in oil reservoirs, various gases are employed, and reducing the surface tension between the injected fluid and oil constitutes one of the primary mechanisms for increasing oil production. Consequently, the second part of the study investigates the effectiveness of synthesized nanoparticles in enhancing foamability, foam stability, and reducing surface tension for their application in EOR processes, representing a novel exploration in this area. Static tests were employed to determine the initial concentration of JNPs required to generate foam and to measure the height of the resulting foam in a visible foam generator column. Finally, three different gases, including CO_2_, CH_4_, and air, were utilized to examine the impact of gas type on the performance of synthesized JNPs at varying concentrations.

## Materials and methods

### Materials

Due to the favorable characteristics and significant potential of SiO_2_ nanoparticles in EOR processes, SiO_2_ nanoparticles served as the foundation for synthesizing JNPs. (3-Aminopropyl)triethoxysilane (APTS, Molar mass = 161.395 g/mol, Density = 0.77 g/cm^3^), Hexamethyldisilazane (HMDS, Molar mass = 221.372 g/mol, Density = 0.946 g/cm^3^), and commercial SiO_2_ nanoparticles with an average diameter of 30 nm were procured from Sigma Aldrich Company. Various materials were utilized in the synthesis process, including solid paraffin wax (CAS number = 8002-74-2, Melting point = 58–62 °C, Density = 0.82 g/cm^3^ at 20 °C) obtained from Tetra-Chem Company. Methanol (CH_3_OH) and Chloroform (CHCl_3_) were provided by Merck Company. All chemicals used were of analytical grade and employed without further modification. Additionally, deionized (DI) water was sourced from Zolal Company.

### Synthesis of JNPs

In this study, the Pickering emulsion method was employed to prepare asymmetric SiO_2_ JNPs. This method is widely recognized for its utility in synthesizing a diverse range of JNPs characterized by small size, controllable morphology, and distinct shapes and compositions^43^. To facilitate the Pickering emulsion, paraffin wax was utilized as the oil medium. Additionally, two different silane agents, namely APTS and HMDS, were employed to confer hydrophobic properties to the surface of SiO_2_ nanoparticles. In the process of creating an oil-in-water Pickering emulsion, bare SiO_2_ nanoparticles were adsorbed onto the paraffin wax/water interface under high temperature conditions. Subsequently, the solution was rapidly cooled to solidify the paraffin wax phase, effectively immobilizing the SiO_2_ nanoparticles at the interface. Subsequent modification of the SiO_2_ nanoparticles was achieved through the application of silane agents. Notably, due to the protective layer of wax, only the exposed surface of the SiO_2_ nanoparticles could be modified^44^. One noteworthy advantage of this method is its versatility, allowing for the synthesis of various JNPs by simply altering the surface modifier agent. The following provides a detailed description of the JNP synthesis method employed in this study.

**Figure 1:**
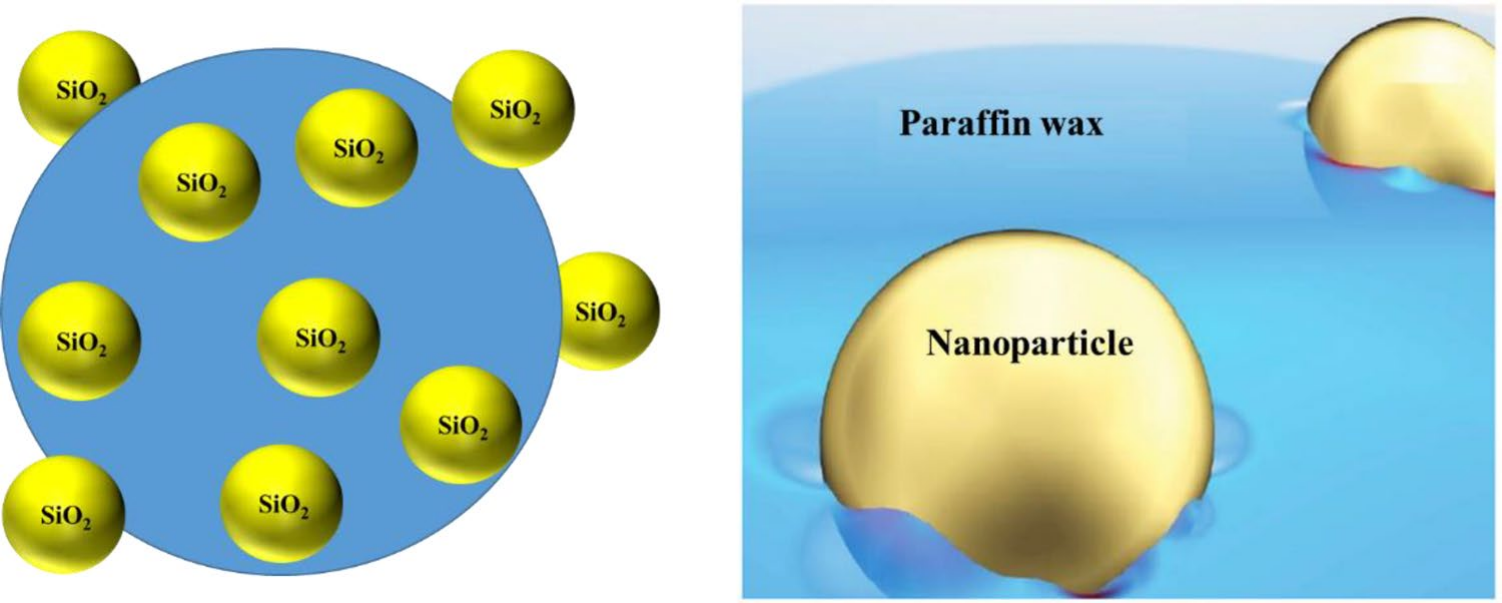
Schematic diagram of nanoparticle coating using paraffin wax.

Initially, 100,000 ppm of SiO_2_ nanoparticles were subjected to sonication in 100 mL of DI-water for 1 h at maximum power. Subsequently, the SiO_2_ nanoparticles were required to adhere to the paraffin wax. To achieve this, solid paraffin wax (weighing 10 times that of the SiO_2_ nanoparticles) was slowly introduced into the prepared solution. The resulting mixture was stirred on a heated stirrer at 110 °C and 400 rpm for a duration of 3 h. Following this, the product was rapidly cooled in an ice bath to prevent the separation of nanoparticles from the paraffin wax, as depicted in Fig. 1. During this stage, the nanoparticles became distributed on the surface of the paraffin wax, with one side of them being coated with paraffin wax.

**Figure 2:**
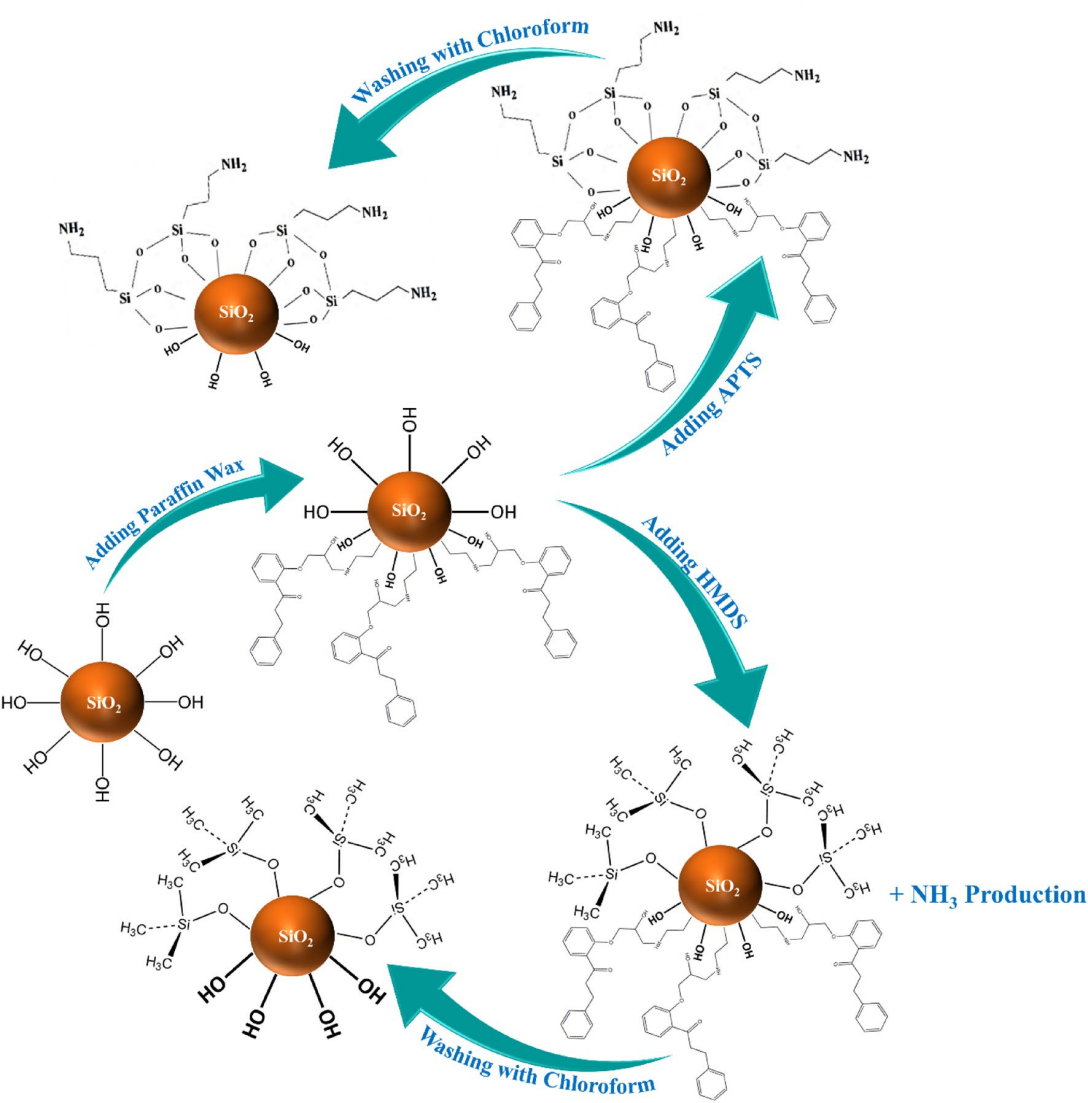
Schematic illustration of SiO_2_ JNPs preparation.

In the subsequent step, two super hydrophobic silanes were employed to coat the exposed surface of SiO_2_ nanoparticles, those not encapsulated within the paraffin. This was carried out to determine the most suitable silane for synthesizing SiO_2_ JNPs. To this end, a 100 mL suspension of paraffin-coated nanoparticles was subjected to centrifugation at 10,000 rpm for 30 min to remove excess liquid from the coated SiO_2_ nanoparticles. Subsequently, the resulting product was dissolved in a 20 mL methanol solvent. Next, 2 mL of either APTS or HMDS silane was added dropwise to the solution, and the mixture was stirred for 12 h in the case of APTS and 2 h for HMDS. It's important to note that the HMDS coating process generated NH_3_ gas as a byproduct. The sample was then centrifuged for 20 min to separate the methanol from the coated nanoparticles. Following this, 5 mL of chloroform was introduced to dissolve the paraffin wax produced during the process. This step was repeated to ensure the complete removal of paraffin wax from the surface of the nanoparticles. Finally, the precipitated products were dried in an oven at 100 °C for 12 h. The schematic of the SiO_2_ JNPs synthesis procedure is illustrated in Fig. 2.

### Characterization

The synthesized products underwent several analyses for characterization. Fourier-transform infrared spectroscopy (FTIR, PerkinElmer Spectrum Version 10.03.06) was utilized to examine the bonding status and functional groups of the coating materials on the surface of the Synthesis JNPs. This analysis was conducted at room temperature within the 4000–400 cm^−1^ range. Dynamic light scattering (DLS, Brookhaven) analysis was employed to determine the hydrodynamic size distribution of the produced particles. In this method, the hydrodynamic diameter of the particles is measured. It's worth noting that due to partial agglomeration of the particles, the obtained size may be larger than their actual size. To reduce agglomeration, synthesized nanoparticles were dispersed in 10 mL of DI-water (at a concentration of 1 ppm) through sonication for 30 min. scanning electron microscopy (SEM) was used to examine the shape and morphology of the produced nanoparticles. To avoid the presence of any agglomerated particles in the captured images, the solution containing synthesized nanoparticles was appropriately diluted. A Zeta potential analyzer device (Zetasizer Nanoseries, Malvern) was employed to measure the surface charge of nanoparticles at room temperature. Zeta potential analysis assesses the repulsion/attraction forces between particles and provides insights into their colloidal stability in a solution.

### Foam stability analysis

**Figure 3:**
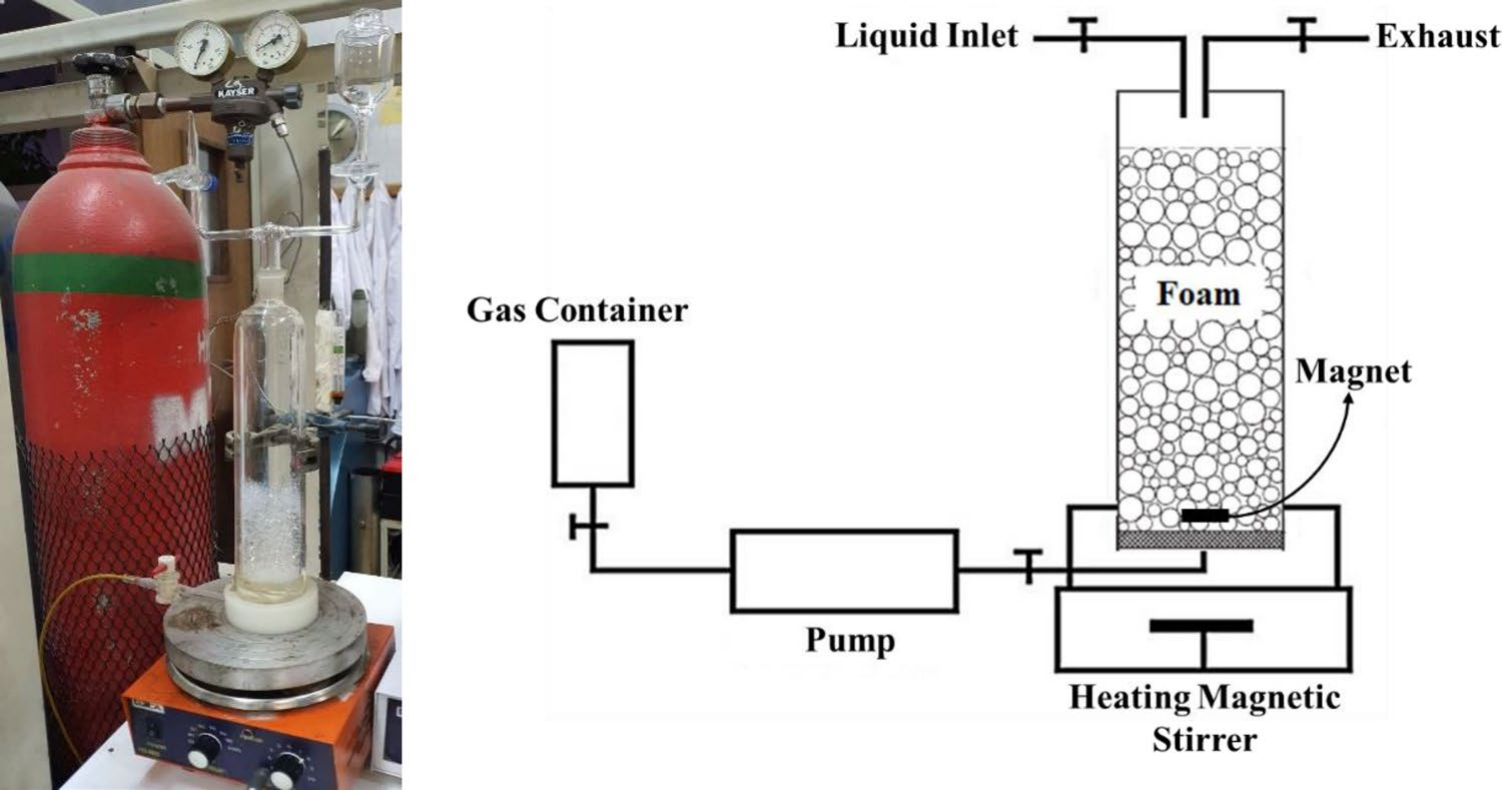
Schematic diagram of the foam stability experimental setup.

The analysis of foam stability assessed the suitability of the synthesized JNPs for use in EOR operations^45^. To this end, various solutions of these nanoparticles at different concentrations were prepared. Figure 3 provides a schematic representation of the experimental setup used to evaluate foam stability. In this procedure, a specific quantity of synthesized nanoparticles was dissolved in 100 mL of DI-water using sonication. Subsequently, these prepared solutions were introduced into the visualization cell, into which three different gases, namely CH_4_, CO_2_, and air, were injected using a pump. The stability of the resulting foam was gauged by monitoring the percentage changes in the height of the generated foam within the foam-generating cell over time. In these analyses, alterations in the foam column height and the extent of stability were measured comprehensively, starting from the moment gas injection ceased and the chamber was sealed, continuing until the foam generated by the nanoparticles had dissipated. All foam stability analyses were conducted under ambient temperature and pressure conditions.

### Surface tension measurement

Surface tension is a crucial parameter for observing the behavior of a fluid at the interface of two phases. In this context, surface tension tests were conducted to investigate the interactions at the gas/water interface in the presence of nanoparticles. Initially, samples were prepared at various nanoparticle concentrations in DI-water. Subsequently, the density of each heterogeneous solution was measured using a pycnometer, and the surface tension of the solution was determined using the pendant drop method. These experiments were conducted under different gas atmospheres, specifically air, CO_2_, and CH_4_, while maintaining ambient pressure and temperature conditions.

## Results and discussion

### Characterization of synthesized JNPs

**Figure 4:**
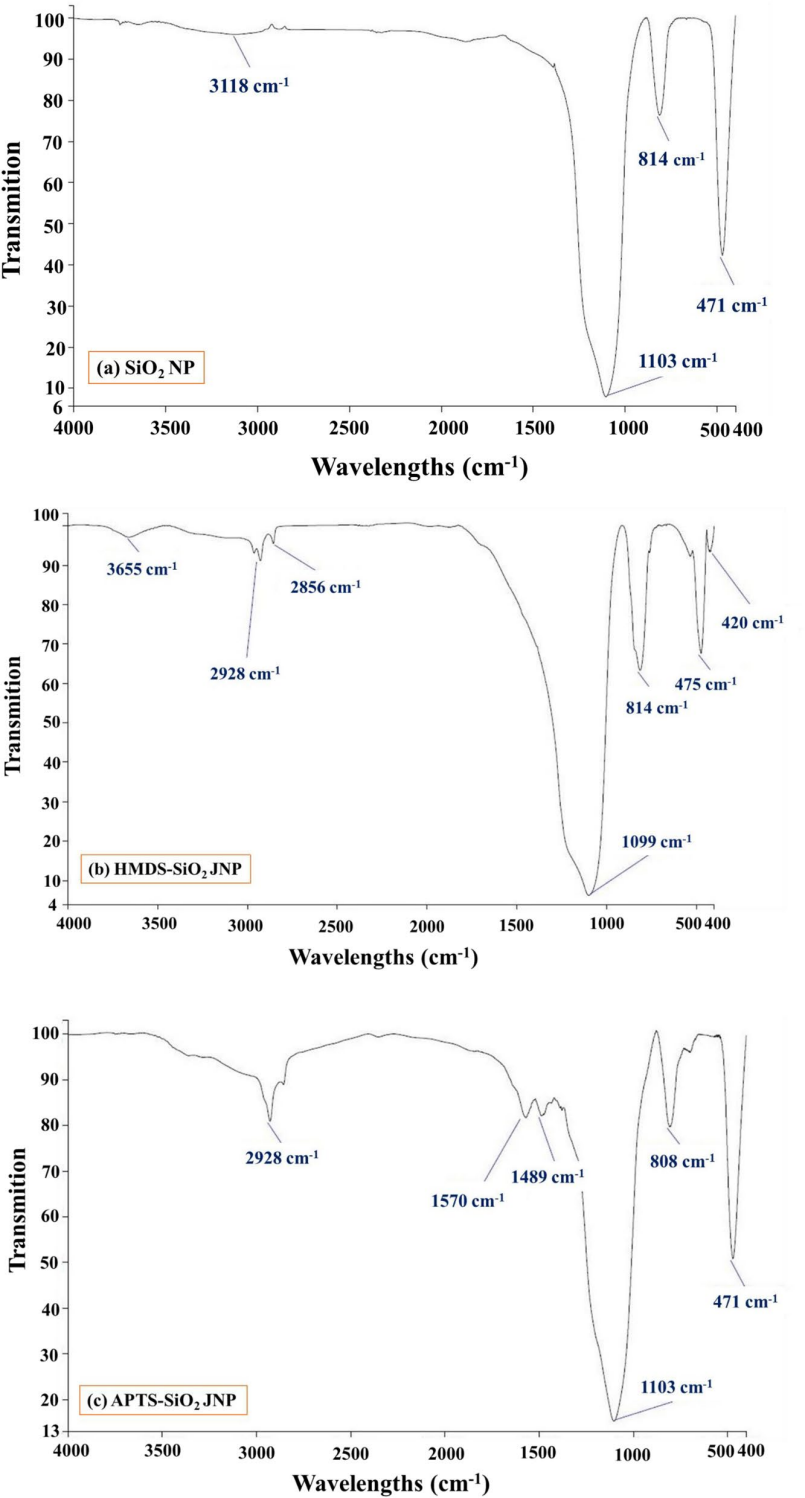
The IR spectra of the bare and SiO_2_ JNPs.

FTIR analysis was conducted to confirm the presence of HMDS and APTS functional groups on the surface of the synthesized JNPs. Figure 4 illustrates the spectra of both bare and SiO_2_ JNPs. In the IR spectra presented in Fig. 4a–c, several key peaks were observed. The peaks at 471–474 cm^−1^, 808–813 cm^−1^, and 1103 cm^−1^ correspond to the Si–O, Si–OH, and Si–O–Si bands in SiO_2_ nanoparticles, and these are consistent across all curves. The broad peaks in the 2800–3400 cm^−1^ range are associated with the stretching vibration of the O–H band. Peaks at 2856 and 2928 cm^−1^ are linked to the C–H stretching bonding of Si–CH_3_ resulting from the reaction with HMDS. Additionally, the absorption peak around 419 cm^−1^ confirms the presence of an HMDS agent on the surface of the synthesized nanoparticles^46^. Following the reaction of SiO_2_ nanoparticles with APTS, peaks around 1489 cm^−1^, 1570 cm^−1^, and 2928 cm^−1^ are observed, which can be attributed to the N–O, N–H, and C–H bands, respectively. These peaks provide evidence for the presence of APTS on the surface of the SiO_2_ nanoparticles^47^.

**Figure 5:**
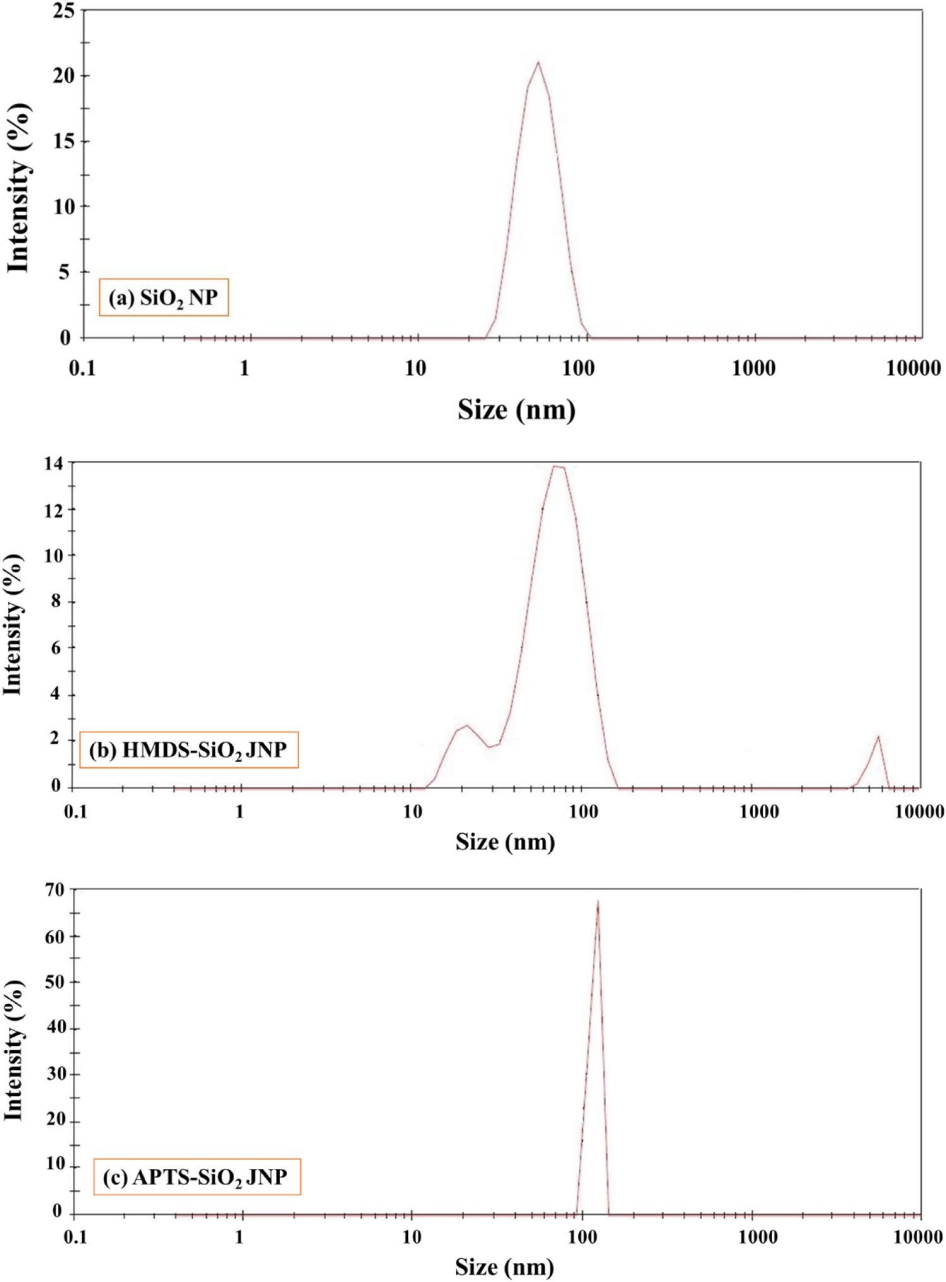
DLS analysis results of synthesized nanoparticles.

Figure 5 displays the DLS results of SiO_2_ nanoparticles before and after modifications. In Fig. 5a, it can be observed that the average hydrodynamic diameter of bare SiO_2_ nanoparticles was approximately 30–50 nm. The laser light diffraction spectrum in this figure encompassed a range of nanoparticles spanning from 15 to 100 nm. Following the surface modification with HMDS, as depicted in Fig. 5b, the average hydrodynamic diameter of SiO_2_ JNPs measured between 70 and 80  nm. In this graph, the smaller peak corresponds to bare SiO_2_ nanoparticles that did not undergo the reaction with HMDS. Conversely, the larger peak is attributed to nanoparticles that were either strongly agglomerated or not entirely separated from the wax paraffin. This observation suggests that a significant proportion of the bare SiO_2_ nanoparticles had indeed reacted with the HMDS silane agent. Figure 5c illustrates the DLS analysis for APTS-SiO_2_ JNPs. It is evident that the particle sizes are very consistent with each other, with the average hydrodynamic diameter of these nanoparticles falling within the range of approximately 100 nm. This also indicates that the reactivity of bare SiO_2_ nanoparticles with APTS silane and their separation from the wax paraffin using a chloroform solution were achieved more effectively.

**Figure 6:**
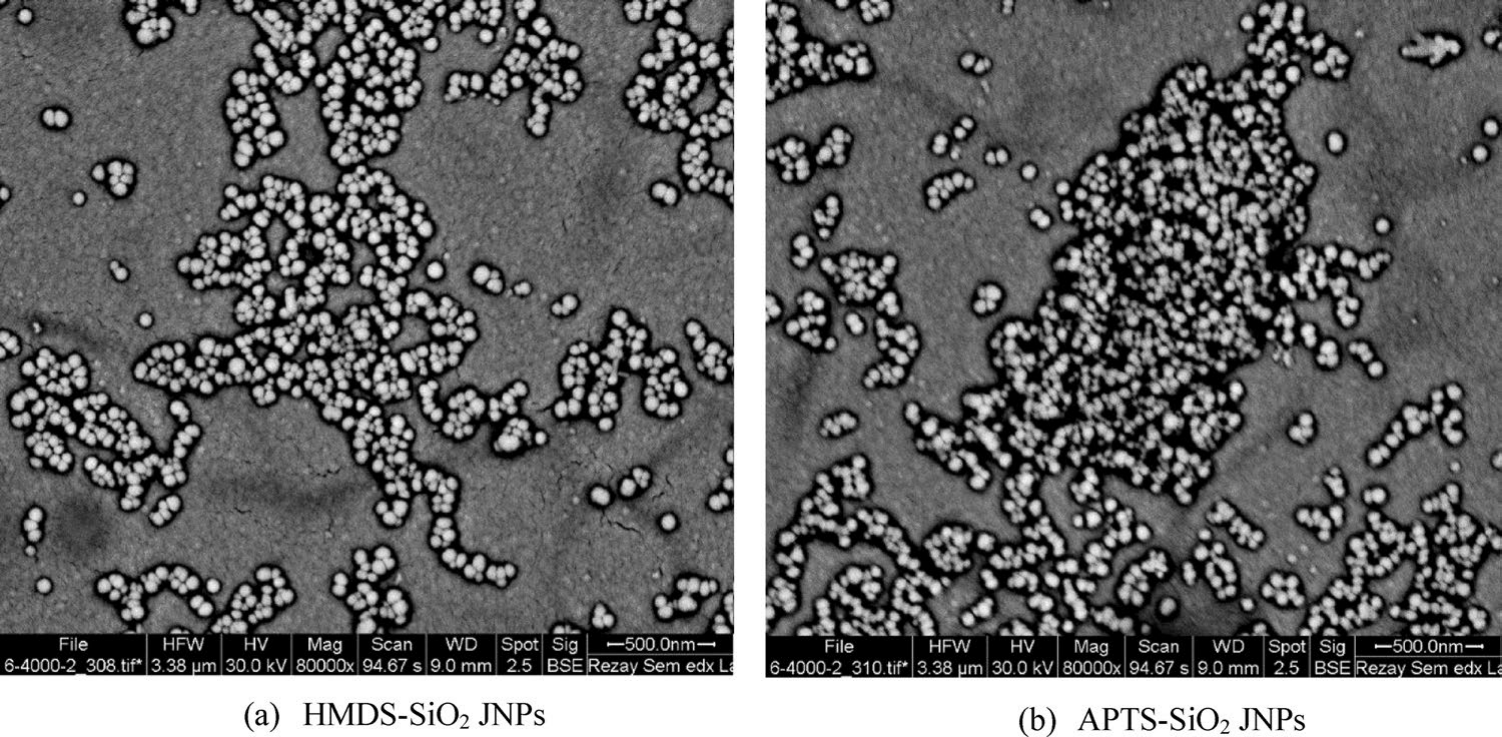
The FESEM images of synthesized SiO_2_ JNPs (the enlargement scale is 80,000×).

Figure 6 presents a representative FESEM image of the synthesized SiO_2_ JNPs. This image illustrates that all the synthesized SiO_2_ JNPs exhibited an approximately spherical shape. Consequently, it can be deduced that the use of silane agents did not have a notable impact on the morphology of the bare SiO_2_ nanoparticles. Additionally, the average size of these nanoparticles was estimated to be less than 100 nm. Notably, the HMDS-SiO_2_ JNPs displayed a more favorable distribution, and they exhibited less agglomeration in comparison to the APTS-SiO_2_ JNPs. While the surface coverage of nanoparticles with a layer of silane agents did lead to an increase in their size, it also enhanced the resistance of these particles for use in acidic and alkaline environments^48^.

It's worth noting that the stability of JNP-stabilized gas foam is significantly influenced by the size of the synthesized JNPs. Reducing nanoparticle size is a critical parameter that can enhance foam stability, increase apparent foam viscosity, improve nanoparticle catalytic performance, and reduce the likelihood of sedimentation within the pores of the reservoir rock. Smaller nanoparticles tend to be readily absorbed at the gas–liquid interface due to their greater diffusion and higher surface-to-volume ratio^49^. Conversely, larger nanoparticles can contribute to foam stability by delaying the liquid drainage process when they are positioned within the lamella and at the plateau border^50^. Therefore, it is reasonable to assume that there exists an optimal size for JNPs that maximizes foam stability. This aspect should be investigated in future research.

Silane compounds consist of relatively long-chain molecules. While these compounds can effectively modify the surface of nanoparticles and enhance their colloidal stability, they generally lead to an increase in the size of nanoparticles compared to materials that employ an electrostatic stabilization mechanism. Consequently, it is recommended that, to maintain the small size of JNPs and establish an appropriate balance between hydrophilicity and hydrophobicity on the surface of SiO_2_ nanoparticles, acid agents such as citric acid or ascorbic acid be considered for the synthesis of SiO_2_ JNPs.

### Zeta potential analysis

Zeta potential is a crucial parameter for assessing the stability of nanoparticles within a colloidal solution. The magnitude of the Zeta potential value reflects the ability of electrostatic repulsion forces between neighboring particles to disperse them within a solution. As such, Zeta potential is directly correlated with the stability of particles in a colloidal solution^51^. Zeta potential analysis was conducted and the results, measured in millivolts (mV), are presented in Table 1 to assess the colloidal dispersion stability of both synthesized coated and uncoated SiO_2_ nanoparticles.

**Table 1:** Results of the Zeta potential analysis.

Sample	Zeta potential (mV)	Stability
Bare SiO_2_ nanoparticles	− 15.3	Moderate stability
HMDS-SiO_2_ JNPs	− 28.5	Good stability
APTS-SiO_2_ JNPs	− 11.5	Incipient instability

The Zeta potential of bare SiO_2_ nanoparticles was measured at − 15.3 mV, which aligns with their apparent stability. After coating the SiO_2_ nanoparticles with HMDS, the Zeta potential of the coated nanoparticles increased to − 28.5 mV. During the silanization process, the surface of the nanoparticles became densely populated with amino group molecules, including the –NH_2_ functional group, leading to a notable increase in the negative charge on the nanoparticle's surface. This enhancement in surface charge contributes to improved nanoparticle dispersibility and stability in aqueous solutions, primarily through a steric stabilization mechanism. Consequently, repulsive forces between the particles are heightened, resulting in enhanced particle stability and reduced agglomeration in the solution^52^.

Conversely, the Zeta potential of coated SiO_2_ nanoparticles with APTS was measured at − 11.5 mV, signifying a slight reduction in particle stability. This decrease can be attributed to the presence of –NH_2_ groups within the structure of SiO_2_ JNPs, which leads to an increase in their hydrodynamic diameter. As a result, the size of SiO_2_ particles experiences a minor increase after being coated with APTS. This phenomenon necessitates a greater force to induce particle sedimentation and deposition in the solution, thereby reducing nanoparticle stability.

### Effect of JNPs dispersed in different gas on the surface tension

**Figure 7:**
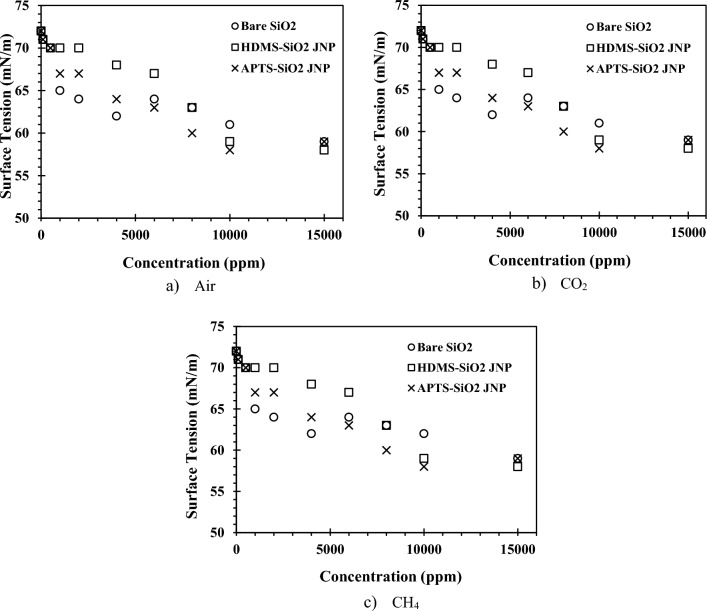
The effect of the nanoparticle concentration on the surface tension the in the presence of different gases.

Nanoparticles play a crucial role in enhancing oil recovery from reservoirs by lowering the surface tension between the injection fluid and the oil. The importance of surface tension stems from its direct impact on the stability of the interface between two phases^53^. Given that the choice of gas in an EOR operation varies depending on the type of oil reservoir and can influence the surface tension of the two phases^54^, we employed three different gases: air, CO_2_, and CH_4_. Figure 7 illustrates the influence of nanoparticle concentration on surface tension in the presence of these different gases.

The surface tension of water without nanoparticles measured between 71 and 72 mN m^−1^. Upon introducing bare SiO_2_ nanoparticles into the liquid phase, surface tension remained relatively unchanged at low nanoparticle concentrations. Typically, nanoparticles tend to establish an interface between water and gas, reducing the interfacial tension between the two phases. However, due to the strong hydrophilic nature of SiO_2_ nanoparticles' surfaces, they are present at the gas–liquid interface in small quantities and do not strongly adhere to it. Consequently, their impact on reducing interfacial tension is limited. As nanoparticle concentration in DI-water increased, the likelihood of nanoparticles being present at the interface also increased. Consequently, more particles could participate at the gas/liquid interface, hindering bubble coalescence and gradually affecting the interfacial surface, leading to reduced surface tension^55^. Consequently, the surface tension of the nanofluid fluctuated between 60 and 65 mN m^−1^ from a concentration of 1500 ppm onwards.

In contrast to bare SiO_2_ nanoparticles, JNPs had hydrophobic surfaces, causing them to predominantly reside at the interface of the two phases. The results demonstrated that HMDS-SiO2 and APTS-SiO_2_ JNPs had a similar effect on reducing surface tension between the two phases in the presence of air. This similarity arises from the strong hydrophobic properties of both synthesized HMDS-SiO_2_ and APTS-SiO_2_ JNPs, with only a small portion of the nanoparticle surface exhibiting hydrophilic characteristics. This amphiphilic structure enables these JNPs to occupy the interface between the two phases to a greater extent than bare SiO_2_ nanoparticles, resulting in a modest reduction in interfacial surface tension (approximately 1–2 mN m^−1^ compared to bare SiO_2_ nanoparticles). All three types of nanoparticles were able to reduce surface tension from 71 to 58–59 mN m^−1^ at 25 °C. Indeed, the balance between hydrophobic and hydrophilic characteristics (amphiphilic structure) plays a crucial role in the ability of JNPs to reduce surface tension. APTS and HMDS compounds, serving as appropriate silane coupling agents, are widely utilized grafting agents to enhance the interfacial behavior of inorganic oxides like SiO_2_. Silanization is a rapid process in its early stages but requires a substantial amount of time to complete the reaction. Hence, controlling the kinetics of silanization is crucial for regulating the surface modification process during JNP synthesis^48,56^.

While different gases had varying effects on surface tension, a consistent trend was observed in both CO_2_ and CH_4_ atmospheres. This similarity can be attributed to the low solubility of these gases in water at atmospheric pressure, as well as the organic nature of CH_4_ gas^42^. In this research, surface tension measurements conducted at atmospheric pressure revealed that CO_2_ gas only dissolves in water to a limited extent, whereas CH_4_, being an organic gas with a fundamentally different nature, does not dissolve in water. Consequently, the interaction between CO_2_ gas and the water droplet's surface resulted in a slight reduction in surface tension compared to the conditions under air^42^.

### Effect of optimal concentration of nanoparticles on foam stability in the presence of different gases

**Figure 8:**
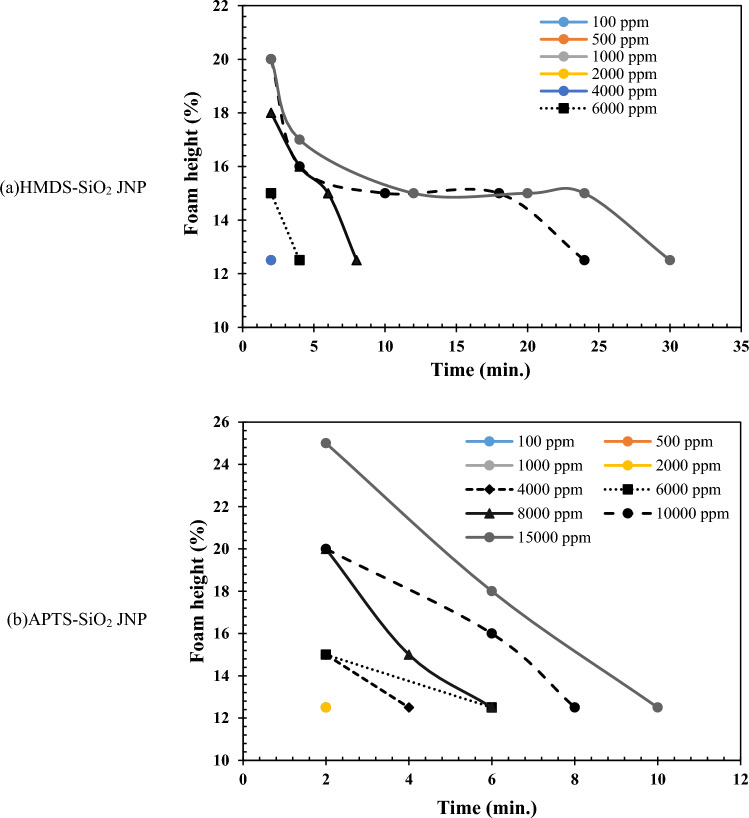
The foam stability analysis results of JNPs in the presence of air.

Surfactants alone are insufficient to stabilize foams under challenging reservoir conditions, such as adsorption on rocks, mixing with oil, emulsion formation, and foam instability at high temperatures. Nanoparticles are expected to have the capability to stabilize foam under ambient conditions. The mechanism of nanoparticle placement is quite similar to that of surfactants in the formation of a thin layer at the water–gas interface^57^. Nanoparticles, being smaller than surfactants and benefiting from the Gypsum–Marangoni effect, possess more surface area and experience enhanced electrostatic repulsion forces between similarly charged particles within the thin water layer. These factors prevent lamellae collapse and promote foam stability^58^. Consequently, JNPs, sharing similarities with surfactants in nature^59^, have been employed in this study. Foam stability analyses were conducted with nanoparticles at various concentrations. Bare SiO_2_ nanoparticles are incapable of stabilizing lamellae due to their high hydrophilic properties and lack of a hydrophobic hydrocarbon chain necessary for forming weak bonds with the gas phase. Therefore, foam stability tests were exclusively conducted with the synthesized JNPs. It was also anticipated that the type of gas could potentially influence foam stability^60^. To investigate this hypothesis, nanoparticles were used at different concentrations under various gas atmospheres to determine the optimal nanoparticle amounts for dispersion in different gases. Figure 8 presents the results of foam stability analyses of synthesized JNPs in the presence of air.

**Figure 9:**
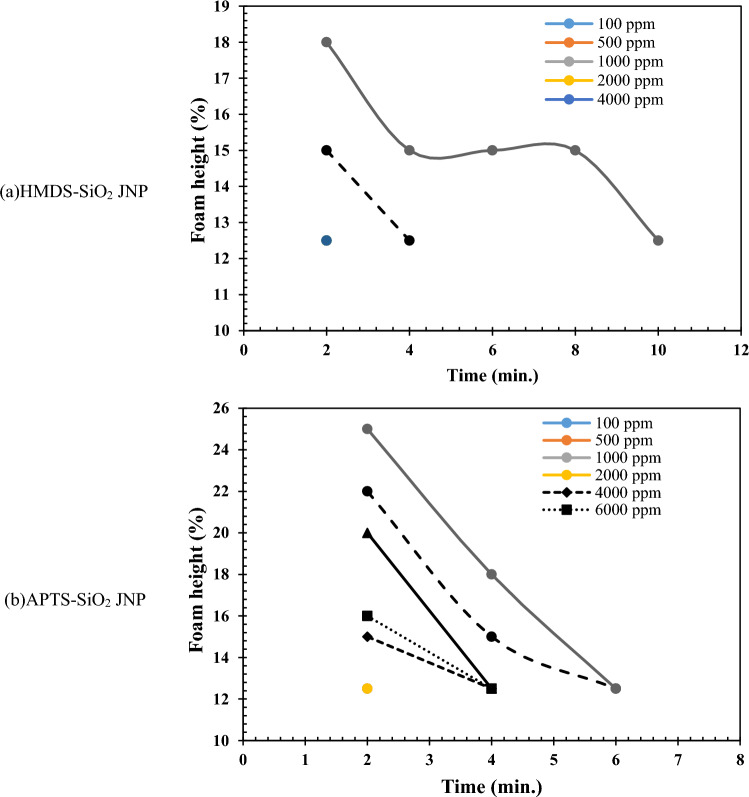
The foam stability analysis results of JNPs in the presence of CO_2_.

As observed, HMDS-SiO_2_ JNPs did not significantly generate or stabilize foam in an air atmosphere. There was no noticeable change in foam height or production until the concentration reached 4000 ppm. However, as the concentration increased from 6000 to 15,000 ppm, the stability time improved from 2 to 28 min. Although the best results were achieved at 15,000 ppm, the optimal concentration for economic considerations was determined to be 10,000 ppm. In contrast to HMDS-SiO_2_ JNPs, APTS-SiO_2_ JNPs exhibited foam stability at a lower concentration, specifically 4000 ppm, with a stability time of 2 min. The balance between the hydrophobic and hydrophilic properties and the presence of amino groups on the surface of these nanoparticles facilitate their dissolution in water, imparting a slightly alkaline property to the solution. This slight alkalinity can have a minor impact on foam stabilization by reducing the interfacial tension^61^. The maximum foam stability, approximately 8 min, was observed at 15,000 ppm. Consequently, it can be inferred that APTS-SiO_2_ JNPs exhibited higher foamability than HMDS-SiO_2_ JNPs, while HMDS-SiO_2_ JNPs demonstrated approximately threefold higher foam stability. The results of foam stability analysis for synthesized JNPs in the presence of CO_2_ gas are presented in Fig. 9.

**Figure 10:**
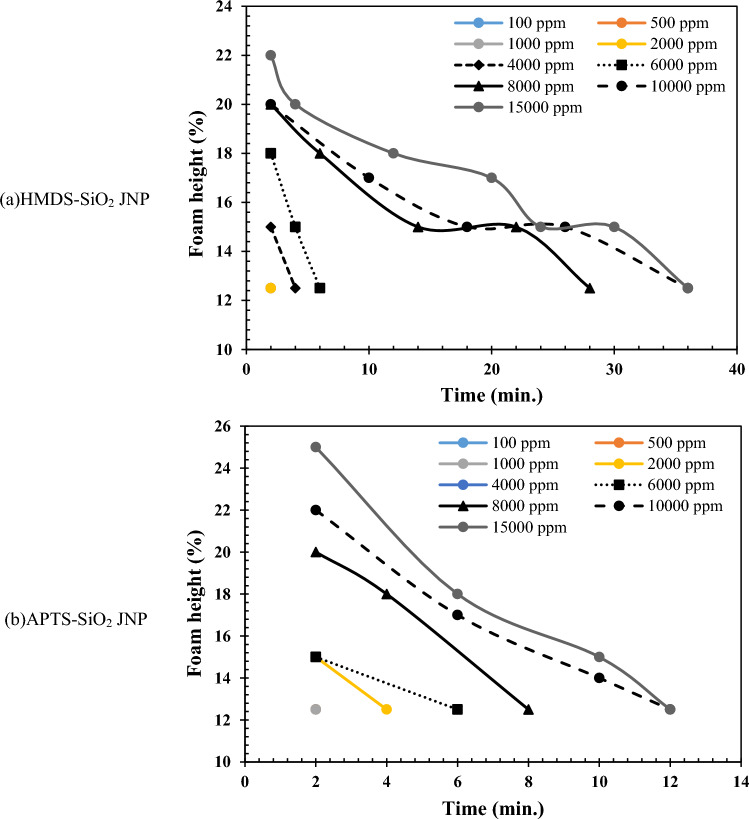
The foam stability analysis results of (**a**) HMDS and (**b**) APTS JNPs in the presence of CH_4_.

In contrast to the air atmosphere, the presence of CO_2_ negatively impacted the foamability achieved using the synthesized JNPs. As depicted, the HMDS-SiO_2_ JNPs exhibited limited ability to generate and stabilize foam in the CO_2_ atmosphere due to their pronounced hydrophobic nature. Only at two concentrations, 10,000 and 15,000 ppm, was some foam produced, and it displayed low stability, with a maximum of 8 min. Conversely, in the case of APTS-SiO_2_ JNPs, there was a modest improvement in foamability, with partial foam formation starting at a concentration of 4000 ppm. However, the foamability and foam stability of these JNPs were lower compared to HMDS-SiO_2_ JNPs. At a concentration of 15,000 ppm, the final stability was only 6 min. The results of foam stability analysis for synthesized JNPs in the presence of a CH_4_ atmosphere are illustrated in Fig. 10.

Figure 10 illustrates that the CH_4_ gas atmosphere was favorable for foamability when using the synthesized JNPs. Due to the hydrophobic nature of HMDS-SiO_2_ JNPs and the high resemblance of CH_4_ gas to an oil phase, a significant portion of the nanoparticles interfaced with the fluids, resulting in relatively good foam stability. However, the foam height did not increase substantially. This nanoparticle does not exhibit surfactant-like properties, and foam formation is primarily attributed to the creation of a nanoparticle layer at the fluid interface. Increasing the nanoparticle concentration from 4000 to 15,000 ppm led to an increase in foam stability from 4 to 36 min. The stability mechanism of APTS-SiO_2_ JNPs differed. This type of nanoparticle behaves somewhat similarly to surfactants due to the presence of a hydrocarbon chain. However, it generally has limited ability to stabilize the water–gas interface. The extent of foam generation and foam stability with these JNPs was lower compared to HMDS-SiO_2_ JNPs. Partial foamability was observed with this nanoparticle starting at a concentration of 2000 ppm. The final stability of the produced foam using this nanoparticle was achieved at a concentration of 15,000 ppm, with stability lasting for 10 min.

The summary of foamability and foam stability analysis results for the synthesized JNPs in this research is presented in Table 2. The amphiphilic structure of the synthesized SiO_2_ JNPs enables these particles to be highly effective at the water/gas interface. The presence of JNPs results in thicker lamellae, indicating slower liquid drainage and prevention of bubble coalescence. The greater the balance between the hydrophilic and hydrophobic heads in the amphiphilic structure of SiO_2_ JNPs, the stronger the tendency for these particles to be located at the water/gas interface. In this scenario, a robust layer with high adhesion energy is formed, leading to the production of long-term stable foam. This balance is more pronounced in the amphiphilic structure of HMDS-SiO_2_. Consequently, HMDS-SiO_2_ JNPs exhibit a greater ability to stabilize foam in the presence of various gases compared to APTS-SiO_2_ JNPs.

**Table 2:** The brief results of foamability and foam stability analyses for synthesized JNPs.

	HMDS-SiO_2_ JNP			APTS-SiO_2_ JNP		
	Foamability con. starting (ppm)	Maximum foam height (%)	Maximum foam stability (min.)	Foamability con. starting (ppm)	Maximum foam height (%)	Maximum foam stability (min.)
Air	4000	20	30	2000	25	10
CO_2_	8000	18	10	2000	25	6
CH_4_	2000	22	36	1000	25	12

## Conclusions

The primary objective of this study was to assess the effectiveness of JNPs in stabilizing foam for EOR processes. To achieve this goal, two types of JNPs were synthesized by coating SiO_2_ nanoparticles with two different silanes: HMDS and APTS. The synthesized JNPs were characterized through various analyses, including FTIR, DLS, and SEM. Foam production was conducted using three different gases: air, CO_2_, and CH_4_. The key findings of this study are as follows:

- The average hydrodynamic diameter of HMDS-SiO_2_ and APTS-SiO_2_ JNPs was measured to be between 70–80 nm and 100 nm, respectively.
- HMDS-SiO_2_ JNPs exhibited greater colloidal stability compared to APTS-SiO_2_ JNPs.
- Incorporating bare SiO_2_ nanoparticles into the liquid phase did not significantly alter surface tension at low nanoparticle concentrations. Both HMDS-SiO_2_ and APTS-SiO_2_ JNPs had a similar effect on reducing surface tension in the presence of different gases. All three types of nanoparticles were able to reduce surface tension from 71 to 58–59 mN m^−1^ at 25 °C.
- Bare SiO_2_ nanoparticles lacked the ability to stabilize the lamella. However, covering their surface with silane agents and synthesizing JNPs improved their performance in foam stabilization.
- APTS-SiO_2_ JNPs exhibited better foamability than HMDS-SiO_2_ JNPs, while HMDS-SiO_2_ JNPs demonstrated approximately threefold higher foam stability.
- In a CO_2_ medium, APTS-SiO_2_ JNPs showed superior foamability compared to HMDS-SiO_2_ JNPs. HMDS-SiO_2_ JNPs could only stabilize foam at concentrations of 10,000 and 15,000 ppm, with a maximum stability duration of 8 min. On the other hand, APTS-SiO_2_ JNPs could maintain foam stability for up to 6 min under optimal conditions.
- In the CH_4_ atmosphere, increasing the concentration of HMDS-SiO_2_ JNPs from 4000 to 15,000 ppm resulted in increased foam stability, ranging from 4 to 36 min. In contrast, APTS-SiO_2_ JNPs exhibited a maximum foam stability time of only 10 min at a concentration of 15,000 ppm.

Overall, the study demonstrates the potential of JNPs, particularly HMDS-SiO_2_ JNPs, for enhancing foam stability in various gas environments relevant to EOR processes.

## Data Availability

All data generated or analysed during this study are included in this article. Email for contact: asaeedi@modares.ac.ir.
